# Growing Ultra-flat Organic Films on Graphene with a Face-on Stacking via Moderate Molecule-Substrate Interaction

**DOI:** 10.1038/srep28895

**Published:** 2016-06-30

**Authors:** Ti Wang, Tika R. Kafle, Bhupal Kattel, Qingfeng Liu, Judy Wu, Wai-Lun Chan

**Affiliations:** 1 Department of Physics and Astronomy, University of Kansas, Lawrence, KS 66045, USA.

## Abstract

The electronic properties of small molecule organic crystals depend heavily on the molecular orientation. For multi-layer organic photovoltaics, it is desirable for the molecules to have a face-on orientation in order to enhance the out-of-plane transport properties. However, it is challenging to grow well-ordered and smooth films with a face-on stacking on conventional substrates such as metals and oxides. In this work, metal-phthalocyanine molecules is used as a model system to demonstrate that two-dimensional crystals such as graphene can serve as a template for growing high quality, ultra-flat organic films with a face-on orientation. Furthermore, the molecule-substrate interaction is varied systematically from strong to weak interaction regime with the interaction strength characterized by ultrafast electron transfer measurements. We find that in order to achieve the optimum orientation and morphology, the molecule-substrate interaction needs to be strong enough to ensure a face-on stacking while it needs to be weak enough to avoid film roughening.

Organic photovoltaics (OPV) is one of the contenders for next generation solar cells due to its low cost, flexibility, light weight and ease of fabrication^1^,^2^,^3^. Most organic semiconductors are anisotropic. As a result, the relative orientations among different molecules and between molecules and the substrate play a decisive role in determining the electronic properties and hence the efficiency of the OPV^4^,^5^,^6^,^7^,^8^. In small molecule organic semiconductors, charge transport primarily occurs in the π-stacking direction because of the stronger electronic coupling^9^,^10^. Moreover, the electron transfer rate at heterostructure interfaces depends on the relative orientation between the donor and acceptor molecules. For example, at the copper phthalocyanine (CuPc)/fullerene (C_60_) interface, it is found that the electron transfer rate from face-on CuPc to C_60_ is four times larger than that from edge-on CuPc to C_60_^5^. In OPV applications, because electrons move along the device’s out-of-plane direction, it is desirable to have the π-stacking direction parallel to the surface normal direction^6^. For most planar organic molecules, it means that the molecular plane is needed to be parallel to the molecule-substrate interface (face-on orientation) in order to attain efficient charge transport.

The orientation of small planar molecules on a substrate depends on the interplay between the molecule-substrate and molecule-molecule interactions^11^,^12^,^13^. For weak molecule-substrate interaction (e.g. molecules deposited on SiO_2_), the molecule-molecule interaction dominates and molecules tend to stand up with the π-stacking direction lying parallel to the interface. Although molecules can be grown in a layer-by-layer fashion with a smooth film morphology^14^,^15^, the out-of-plane electronic transport is slow. This edge-on orientation favors applications such as organic field effect transistors^8^,^15^, but it is undesirable for photovoltaics and other opto-electronic applications. On the other hand, for a strong molecule-substrate interaction (e.g. molecules deposited on metal substrates), the molecules have a face-on stacking^16^,^17^,^18^,^19^. However, the strong molecule-substrate interaction often results in a metastable crystal structure. Beyond a few monolayers, the film will relax by forming mounds on the surface. In some cases, the molecules will even switch to a standing-up orientation^20^,^21^. Indeed, it is a challenge to grow well-ordered and smooth organic films with a face-on orientation on conventional metal and oxide substrates^22^. Recently, it is demonstrated that one can circumvent this limitation by using substrate with an “intermediate” molecule-substrate interaction. For example, Si substrate is doped degenerately to reduce the molecule-substrate interaction such that thick face-on oriented films can be grown without significant surface roughening^16^. A few studies show that graphene is a good template for growing uniform organic films with a face-on stacking^23^,^24^,^25^,^26^. However, these studies mainly focus on the structural and electrical characterization of the organic layer grown on graphene. It is unclear how specifically the film morphology depends on the molecule-substrate interaction and why molecular crystals grown on graphene have better morphology compared to that grown on metals^21^ even though both has a face-on orientation.

In this work, ZnPc molecules are used as a model system to investigate the relationship between the morphology, molecular orientation and interfacial electronic properties by systematically varying the molecule-substrate interaction from strong to weak interaction regime. A previous comprehensive study of CuPc grown on graphene^24^ has shown that the morphology and molecule orientation can depend on the film thickness and the growth temperature. In this work, we will fix these other parameters to investigate the effect of the substrate on the film properties. A series of substrates including Cu, Au, graphite, graphene and SiO_2_/Si, were selected to provide progressively weaker molecule-substrate interactions. Compared to previous works which mainly focus on the orientation of molecules on different substrates, we investigate how the initial molecule-substrate interaction would eventually affect the film morphology and roughness when the film becomes thicker. The film morphology and roughness, on top of the molecular orientation, can significantly affect the transport properties and device performance. The orientation of the molecules was determined by the work function of the film measured with ultraviolet photoemission spectroscopy (UPS)^7^,^27^, while the surface morphology was characterized using atomic force microscopy (AFM). Compared to the Cu and Au, ZnPc thin films on the HOPG and graphene show a smoother morphology because the weaker molecule-substrate interaction in the latter allows the films to be grown with a more relaxed structure. However, the presence of step-edges on HOPG surfaces induces preferential nucleation of ZnPc islands at step-edges, resulting in a non-uniform film coverage. This issue can be addressed by using graphene since its atomic flat surface enables the formation of ultra-smooth ZnPc films with uniform coverage.

Furthermore, the interfacial charge transfer from the molecule to the substrate, which reflects the interfacial electronic coupling, is measured by femtosecond (fs) time-resolved photoemission spectroscopy (2PPE). The stronger molecule-substrate interaction at the ZnPc/metal interface compared to that at ZnPc/graphite and ZnPc/graphene interfaces results in a faster charge transfer rate and a spatially larger interaction range. Among all the substrates studied, we find that graphene, which has a moderate interaction with the organic molecules, enables molecules to be grown with face-on orientation while avoiding significant structural relaxation as the film grows thicker.

## Results and Discussion

### Surface Morphology

**Figure 1:**
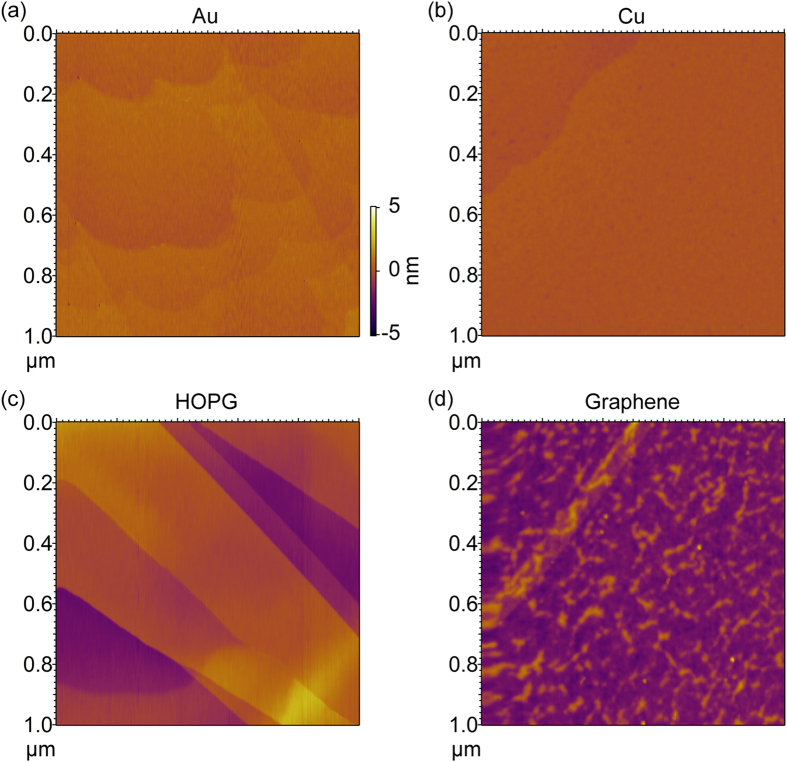
AFM images of the (**a**) Au, (**b**) Cu, (**c**) HOPG, and (**d**) graphene substrates before ZnPc deposition.

All the metal substrates are cleaned in ultrahigh vacuum (UHV) environment using standard sputter and annealing procedures (see Method section). In the case of graphite, the surface is cleaved before subsequent annealing and deposition. The AFM images of the bare substrate are shown in Fig. 1. On all these substrates (Au, Cu, HOPG), the surface is cleaned and has a small initial rms roughness (<0.7 nm). Step-edges on these surfaces can be seen in the AFM images. For the graphene on SiO_2_ sample, the surface appears to have some wrinkles on the surface. It is known that a single layer of graphene can form wrinkles and ripples on its supporting substrate^28^. The small grain size found in CVD graphene^29^ may also result in these initial roughness. A discussion on the origin of this roughness is out of the scope of this work, but we note that the rms roughness of the graphene substrate is still small (0.74 nm).

**Figure 2:**
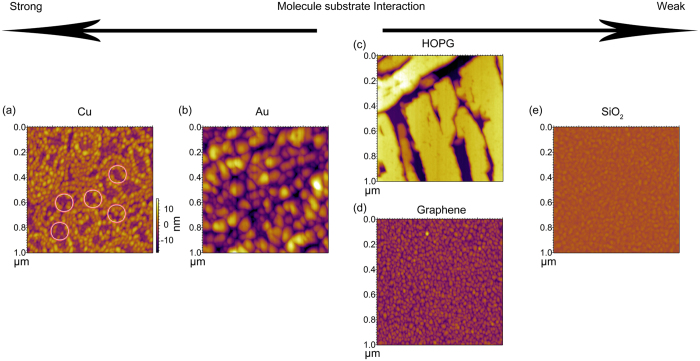
AFM images of 10 nm ZnPc thin films deposited on (**a**) Cu, (**b**) Au, (**c**) HOPG, (**d**) graphene and (**e**) SiO_2_. For comparison, the same color scale is used for all the images.

Figure 2 shows the morphology measured by AFM for 10 nm ZnPc films deposited on various substrates. Images with a larger scan size are presented in the Supplementary information, which show that the morphology is similar at a larger length scale. Similar morphology is observed on different areas of the samples. Moreover, a thickness of 10 nm is chosen because the effects of the substrate on the morphology are clearly manifested at this thickness. At larger thicknesses, other effects such as molecule-molecule interactions or grain coalescence may start to couple to the substrate-effect to affect the film morphology. For example, a recent work^24^ on CuPc films on graphene has found that, at room temperature, the molecules have a face-on orientation at a film thickness of 20 nm, but some molecules switch to an edge-on orientation when the film thickness increases to 50 nm. The switching of the molecular orientation suggest that molecule-molecule interaction dominates at larger thicknesses. Considering this work focuses primarily on revealing how the film morphology depends on the substrate-molecule interaction, a smaller thickness of 10 nm was selected to avoid the complication of molecule-molecule interaction.

**Figure 3:**
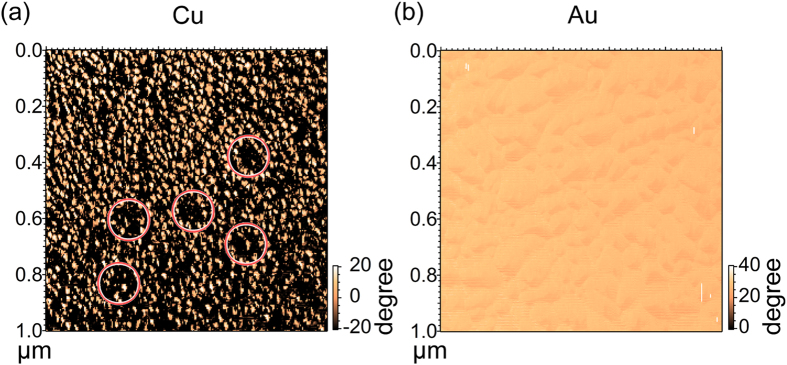
The AFM phase images of ZnPc on (**a**) Cu and (**b**) Au obtained from the same regions shown in Fig. 1a,b, respectively. The red circles in (**a**) are the same positions presented in Fig. 2a, in which the phase angle has consistently smaller value compared to that in other regions.

For the film deposited on Cu(001) (Fig. 2a), many small islands are formed on the surface. The ZnPc film grown on Au(111) surface shows a clear increase in grain size (Fig. 2b). Figure 3 shows the phase images obtained from the same measurement shown in Fig. 2a,b. The ZnPc/Cu shows a much stronger phase contrast than the ZnPc/Au (Fig. 3). The phase contrast is commonly originated from a variation of surface composition or surface adhesion. The correlation between regions with a lower surface height and regions with a smaller phase angle (e.g. areas enclosed by red circles in Figs 2a and 3a) indicates that part of the Cu surface is exposed. In contrast, the phase-image of ZnPc/Au shows a more uniform intensity indicating that the ZnPc on Au has a complete coverage.

A number of studies have been devoted to investigate the interaction between small molecules and metal surfaces^18^,^19^,^30^,^31^,^32^. It is known that organic molecules interact more strongly with Cu than with Au. For example, a recent study shows that when CuPc molecules are deposited on Cu, commensurate islands are formed at monolayer coverage, indicating that molecules are chemisorbed on the surface. For Au surfaces, the CuPc molecules are physisorbed and behave as a two-dimensional gas, which allows the formation of a uniform wetting layer^18^. The stronger molecule-substrate interactions between ZnPc and Cu can reduce the mobility of ZnPc molecules on the Cu surface, which explains the smaller feature size and the discontinuity observed in the ZnPc/Cu samples. Furthermore, our previous study on ZnPc/Au shows that the film has a layer-by-layer growth mode at film thickness <5 nm^33^, but the island morphology observed in Fig. 2b develops only when the film becomes thicker. The AFM images of the 2 nm ZnPc film on Au are shown in the Supplementary information. It shows that most parts of the film are smooth with some small islands starting to nucleate on the surface. This kind of growth mode is typically known as the Stranski-Krastanov (SK) growth^34^. The formation of islands is usually driven by the relaxation of the film stress. In our case, the ZnPc-Au interaction can result in a ZnPc crystal that has slightly different lattice parameters compared to that of the equilibrium structure (i.e. the crystal is strained). The formation of islands at a later stage is evidently driven by structural relaxation.

The films grown on Cu and Au either show an incomplete coverage or an island growth morphology at large film thicknesses. In order to grow ultra-smooth and well-ordered films with uniform coverage, further weakening of the molecule-substrate interaction is necessary. Substrates such as graphite and graphene interact with ZnPc via weaker van der Waal’s interaction. However, the molecule-substrate interaction should still be strong enough to ensure a face-on stacking^35^,^36^,^37^. The surface morphology of ZnPc films grown on HOPG is shown in Fig. 2c. As seen in Fig. 2c, the top of the ZnPc grains are atomically flat, but some deep gaps exist between grains. Comparing Figs 2c and 1c, the shape of the grains correlates with the shape of the atomic terraces on the bare graphite surface. Therefore, the formation of these deep gaps is apparently related to the step edges on the graphite surface. For example, a recent work on ZnPc on Si(111)-Boron surface shows that the ZnPc nucleates preferentially on the step-edges of Si(111) surfaces^16^.

Further improvement in the film morphology requires elimination of the surface step-edges. This motivates us to use graphene as a template for ZnPc growth, which does not contain any step edge on the surface. Fig. 2d shows the AFM image of 10 nm ZnPc deposited on graphene. The ZnPc film covers the substrate uniformly with a low root mean square (RMS) roughness (2.47 ± 0.28 nm). It is not clear whether the small-sized features observed correspond merely to surface roughness or to a film with a small grain size. However, the lateral size of these features is similar to those observed on the bare graphene surface (Fig. 1d). We expect that the quality of the film would be further improved if the graphene does not have those wrinkles observed in Fig. 1d. Note that the surfaces of the ZnPc grains deposited on both graphene and graphite are smooth, but some discontinuities are found in the film on graphite due to the presence of step edges on the bare graphite surface. Based on this observation, it is expected that the morphology of ZnPc films grown on multi-layer graphene should be similar to that on single layer graphene if the top graphene layer is continuous.

One may further reduce the molecule-substrate interaction by using substrates such as SiO_2_/Si. However, it is known that because the molecule-molecule interaction is now stronger than the molecule-substrate interaction, the molecules will have an edge-on (or standing-up) orientation with the fast transport direction parallel to the surface. Indeed, these films usually show extremely smooth morphology because the nucleation of a new layer is a slow process and the growth proceeds with a layer-by-layer mode. Fig. 2e shows the morphology of ZnPc films on SiO_2_/Si with the RMS roughness of 0.78 ± 0.03 nm, which is less than the height (~1.2 nm for the edge-on orientation) of the molecule.

### Molecular orientation

**Figure 4:**
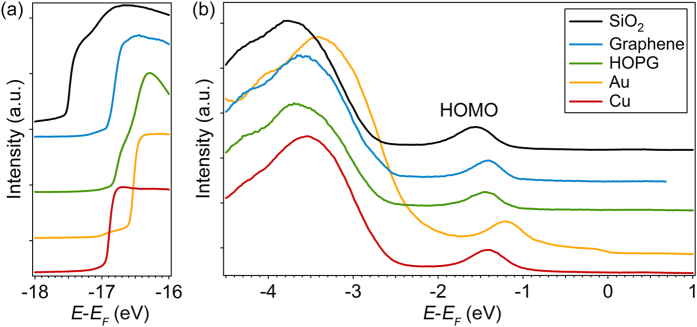
UPS spectra of 10 nm ZnPc thin films on SiO_2_, graphene, HOPG, Au and Cu. The secondary electron cutoff and the HOMO are shown in (**a**) and (**b**) respectively. The UPS spectra are collected with the He-I emission line with photon energy of 21.22 eV.

As mentioned earlier, electronic properties of organic films depend heavily on the orientation of the molecules. Here, the molecular orientation is determined by UPS. Previous works have shown that the ionization potential of molecular crystals depends on the orientations of molecules with respect to the substrates. For pi-conjugated small molecules, films with face-on molecules typically have an ionization potential ~0.5 eV larger than that for films with edge-on molecules^7^,^27^. Therefore, the ionization potentials of ZnPc can be used to determine the orientations of the molecules. Figure 4 shows the UPS spectra of 10 nm-thick ZnPc films deposited on various substrates. The region near the secondary electron cutoff (SECO) and the highest occupied molecular orbital (HOMO) are shown in panel (a) and (b) respectively. The ionization potentials can be calculated from the position of the SECO and the HOMO edge, which are 5.24 eV, 5.23 eV, 5.27 eV and 5.28 eV for 10 nm ZnPc films on Cu, Au, HOPG and graphene respectively. On the other hand, the ionization potential decreases to 4.82 eV for the ZnPc films deposited on SiO_2_/Si. It is known that molecules deposited on SiO_2_ have an edge-on orientation because of the weak substrate-molecule interaction^14^,^15^. The ionization potential of ZnPc films found on other substrates is about 0.4–0.5 eV larger, which indicates that the molecules adopt a face-on structure on Cu, Au, HOPG and graphene. The measured ionization potential is consistent with previous measurements on CuPc thin films^27^, in which CuPc molecules with face-on and edge-on orientations have an ionization potential of 5.15 eV and 4.75 eV respectively. The position of the SECO for the edge-on domains would appear at energy ~0.4 eV lower compared to that of the face-on domains. The sharpness of the SECO and the weak photoemission intensity below the cutoff energy found in ZnPc/graphene indicates that most of the molecules have a face-on orientation. This agrees with a comprehensive X-ray study done by Xiao *et al*.^24^, which shows that CuPc on graphene has a face-on orientation for film thicknesses up to 20 nm.

To further support the UPS results, low-energy electron diffraction (LEED) is done for 10 nm ZnPc on Au and HOPG. The diffraction patterns are shown in the supplementary information, which show that the ZnPc molecules on Au and HOPG have a face-on orientation and consistent with the orientation determined using the ionization potential. Moreover, similar UPS and LEED results can be obtained on different areas of the surfaces, which indicates that the same molecular orientation is found across the film.

**Figure 5:**
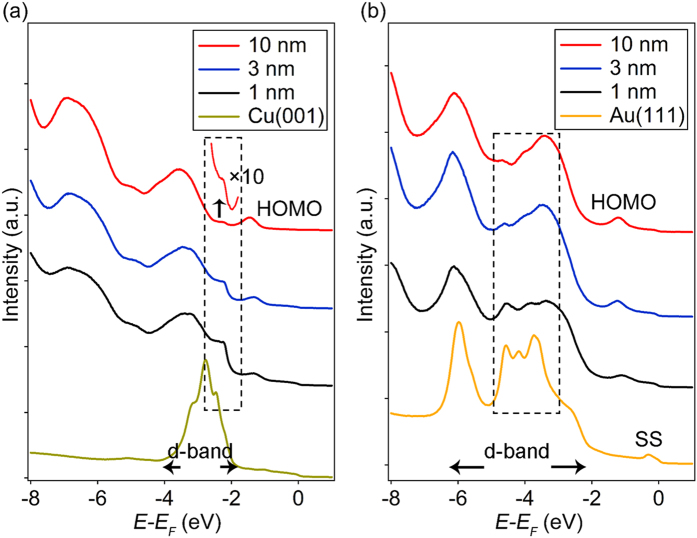
(**a**) UPS spectra of Cu(001), 1, 3 and 10 nm ZnPc thin films on Cu. (**b**) UPS spectra of Au(111), 1, 3 and 10 nm ZnPc thin films on Au. The “SS” label represented the surface state of Au.

In our earlier discussion, it is mentioned that the ZnPc film on Cu does not fully cover the surface, which is also reflected in our surface-sensitive UPS measurements. Figure 5a shows the UPS spectra of Cu(001), 1 nm, 3 nm and 10 nm-thick ZnPc on Cu(001). Since UPS is a surface sensitive technique, the spectra represent signal originated from the film surface. Copper has a d-band peak located between 2 eV and 4 eV below the Fermi level. The lower energy edge (enclosed by the dashed rectangle) of this d-band peak is clearly observed for the 1 nm and the 3 nm ZnPc films. This edge can still be observed in the 10 nm-thick ZnPc film, even though the intensity is much reduced. Therefore, we conclude that a portion of the Cu surface is still exposed for films as thick as 10 nm, which is consistent to the AFM measurement. For comparison, the corresponding spectra for ZnPc films grown on Au(111) are shown in Fig. 5b. Similar to Cu, Au has a d-band peak located at around 2–6 eV below the Fermi level. Some of the feature from the d-band peak can be observed in the spectrum of the 1 nm film (dashed rectangle). However, for larger thicknesses, the spectra resemble to that of the thick ZnPc film. As shown in Fig. 5b, the 3 nm spectrum is almost identical to the 10 nm spectrum. This is consistent to our earlier interpretation that ZnPc forms a complete wetting layer on the Au surface before transitioning to an island growth mode.

### Electronic coupling at the molecule-substrate interface

**Figure 6:**
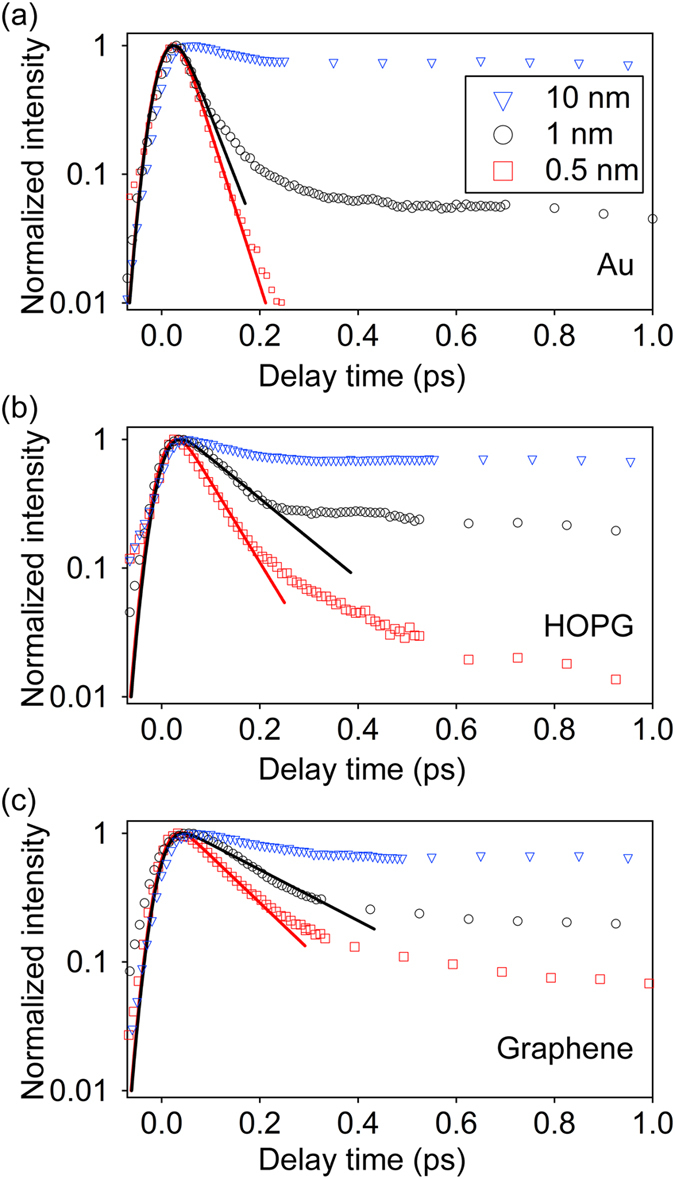
The integrated and normalized photoemission intensity of the ZnPc singlet state as a function of time for 0.5 nm, 1 nm, and 10 nm-thick ZnPc deposited on (**a**) Au, (**b**) HOPG and (**c**) graphene. The initial CT rate is fitted with an exponential function convoluted with the instrumental response function. The fits are shown as solid lines.

Molecular orientation affects the dynamical charge transfer rate at the interface^5^,^6^ because the electronic-coupling between the donor and acceptor molecules depends strongly on their relative orientations. Similarly, a stronger electronic coupling between the substrate and interface should result in a faster interfacial charge transfer rate. Here, we characterize the interfacial charge transfer rate by using 2PPE spectroscopy. We have used this technique previously to characterize the interfacial charge transfer rate for ZnPc molecules deposited on graphene^38^. We note that other similar techniques such as resonant photoemission spectroscopy have also been used to study charge transfer across molecule/metal interfaces^39^. The details of our ultrafast experiment can be found in refs 33,38. Briefly, since 2PPE is surface sensitive, the lifetime of the optically-excited exciton in ultrathin ZnPc layer deposited on various substrates is determined by the charge transfer rate from the molecules to the substrate. Figure 6 shows the integrated photoemission intensity originated from the optically-excited singlet exciton of ZnPc deposited on Au, HOPG and graphene. The photoemission intensity can be interpreted as the population of the singlet exciton located near the surface. The results for three different film thicknesses: 0.5 nm, 1 nm and 10 nm are shown. The dynamics observed for the 10 nm samples on various substrates are almost identical. This is because excitons near the film surface cannot interact with the substrate at this short time-scale and the dynamics represents the intrinsic dynamics of the ZnPc thin film.

For the 0.5 and 1 nm samples, the photoemission intensity shows a more rapid decay. The initial ultrafast decay of the intensity is attributed to the charge transfer from ZnPc thin films to the substrates. After the first few hundreds fs, the intensity decreases at a much slower rate. This is attributed to the relaxation of excited electrons to lower energy traps, which can slower down the electron transfer. The initial decay is fitted with an exponential function (solid lines) convoluted with the finite temporal-width of the laser pulses. The decay times (the inverse of the decay rate) obtained from the fit are summarized in Table 1.

Two main observations can be made. First, the decay times are shorter for Au and progressively become larger for graphite and graphene. In our previous work, we find that the band-alignment at the molecule/substrate interface is similar for graphite and graphene. In both cases, the singlet level is about 0.3–0.4 eV higher than the Fermi level of the substrate^38^. For ZnPc/Au, the singlet level of ZnPc is located at 0.65 eV above the Fermi level of Au^38^. Because of the relative small reorganization energy (0.1–0.2 eV) of ZnPc molecules^40^,^41^, we expect that the electrons will transfer to states in the substrate that have similar energy to the molecular state^42^. In all these substrates, since unoccupied states are available at all energies above the Fermi level, electrons can be transferred directly to states with energies around the singlet level. Therefore, it is not expected that the actual energy alignment would significantly affect the transfer rate. On the other hand, the slower transfer rate for graphene compared to that for graphite can be explained by the reduced dimensionality (from 3D to 2D). It is known that the density of states near the Fermi level in graphene is reduced significantly compared to that of graphite^43^. The smaller number of acceptor states in graphene would result in the lower transfer rate even though the ZnPc/graphite and ZnPc/graphene should have similar interfacial structure. The even faster transfer rate to Au would indicate that the molecule-substrate interaction is stronger for Au as compared to that for graphite and graphene. The very fast charge transfer rate in ZnPc/Au also agrees with previous core-hole clock photoemission spectroscopy studies on CuPc/Au^44^. The stronger molecule/substrate interaction in ZnPc/Au is in accord with the evolution of the surface morphology discussed earlier.

Second, the decay time for ZnPc on Au increases only slightly when the thickness increases from 0.5 to 1 nm. On the other hand, the decay times for ZnPc on HOPG and graphene are almost doubled when the film thickness is increased from 0.5 nm to 1 nm. This result implies that Au has a spatially larger molecule-substrate interaction range with ZnPc molecules as compared to that of graphite and graphene. Since the height of lying down ZnPc molecules is ~0.32 nm^45^, the interaction range between ZnPc and Au can extend up to a few molecular layers. The electronic wavefunction for states originated from metal substrates often has a tail that extends above the surface^46^, which would result in a long range interaction between ZnPc and Au. The larger interaction range would induce a crystal structure in ZnPc that is slightly deviated from the crystal structure for thicker films. This explains why the ZnPc on Au follows the classical SK-growth mode. When the initial wetting layer reaches a certain critical thickness, structural relaxation drives the nucleation of grains with a more stable structure. This results in the formation of islands at larger film thicknesses.

Finally, we like to comment that the samples used to determine the charge transfer rate is either 0.5 nm or 1 nm thick, which should allow them to probe exclusively the molecule-substrate interaction. Any difference in the long-range structural order and the morphology of the ZnPc films as the film subsequently grown thicker cannot affect the measured charge transfer rate. Moreover, monolayer of planar metal-phthalocyanines has very similar orientation and crystal structure when they are deposited on Au(111)^47^ and HOPG surfaces^48^. Therefore, the difference in transfer rate is not likely to be caused by a difference in the molecular orientation.

## Conclusions

In conclusion, by varying the molecule-substrate interaction that covers the strong (Cu, Au), intermediate (graphite, graphene) and weak (SiO_2_) interaction regime, the change in the surface morphology and molecular orientation of ZnPc films deposited of these substrates has been investigated systematically. It has been found that in the intermediate interaction regime, the ZnPc molecules can maintain the face-on stacking, while the surface morphology is significantly improved as compared to those deposited on metal substrates of a strong molecule-substrate interaction. The variation of the molecule-substrate interaction is determined independently by measuring the electron transfer rate between the molecules and the substrate. On metal substrates, a stronger electronic coupling and a larger interaction range are found as compared to graphite and graphene. Our work illustrates the importance in controlling the molecule-substrate interaction in achieving a desirable morphology for the organic films. In this work, film thickness up to 10 nm is investigated, but it is expected that an initial smooth film morphology and the layer-by-layer growth mode observed in ZnPc on graphene should suppress film roughening and nucleation of grain with different orientations as the film grows thicker. Growing smooth and continuously organic films with a face-on stacking is important for photovoltaics applications. The current work shows that graphene and related 2D materials can be good templates for growing such organic films.

## Method

### Sample Preparation

ZnPc thin films were deposited on Cu(001), Au(111), HOPG, graphene and SiO_2_/Si by thermal evaporation in an ultrahigh vacuum (UHV) chamber with base pressure <5 × 10^−10^ Torr. The Cu(001) and Au(111) single crystal surfaces were prepared by sputtering with 1 keV Ar ions and annealing at 500 °C repeatedly until a clean surface was obtained. For the HOPG substrate, the surface layer was peeled off with scotch tape to expose a fresh surface before it was introduced into the vacuum chamber. Single-layer graphene was either grown directly on SiO_2_/Si using CVD^49^ or transferred from Cu foil onto SiO_2_/Si using PMMA^50^. No significant difference was observed in the results obtained from the two different graphene. The SiO_2_/Si wafer was clean in methanol and acetone before it was introduced into the vacuum chamber. The HOPG, graphene and SiO_2_/Si samples were annealed in the UHV chamber at 400 °C for 12 hours before deposition. The ZnPc molecules (Luminescence Technology, >99%) were vapor-deposited on different substrates at room temperature at a rate ~0.8–1 Å/ min. Then, the sample was transferred under UHV environment to another chamber with a base pressure <1 × 10^−10^ Torr where UPS and time-resolved 2PPE experiments were performed.

### Sample Characterization

The AFM was done with a Digital Instruments Multimode V atomic force microscope. Tapping-mode was used to avoid surface damages. The UPS experiment was performed with the He-I (21.2 eV) line from a standard gas discharged lamp. For the ultrafast time-resolved measurements, the sample was excited by visible pump laser pulses with wavelength centered at 700 nm. The pulses, which had duration of ~25 fs, were generated from a non-collinear optical parametric amplifier NOPA (Orpheus- N-2H, Light Conversion). The excited electron was ionized by a time-delayed UV probe laser pulse (wavelength ~280 nm) which was generated by frequency-doubling the output of a second NOPA (Orpheus- N-3H, Light Conversion). Both NOPAs were pumped by a Yb:KGW regenerative amplifier running at a rate of 125 kHz (Pharos 10 W, Light Conversion). The kinetic energy of the photoelectron was determined by a hemispherical electron analyzer (SPECS Phoibos 100). Further details of the experimental setup can be found in refs 33 and 38.

## Additional Information

**How to cite this article**: Wang, T. *et al*. Growing Ultra-flat Organic Films on Graphene with a Face-on Stacking via Moderate Molecule-Substrate Interaction. *Sci. Rep.* **6**, 28895; doi: 10.1038/srep28895 (2016).

## Supplementary Material

Supplementary Information

## Figures and Tables

**Table 1:** The decay times of the photoemission intensity for the 0.5 nm and the 1 nm ZnPc films deposited on various substrates.

Substrate	0.5 nm	1 nm
Au	40 ± 10 fs	45 fs ± 10 fs
HOPG	70 ± 10 fs	140 fs ± 10 fs
Graphene	140 ± 20 fs	220 fs ± 20 fs

These decay times are determined by fitting the experimental data shown Fig. 5 with an exponential decay function convoluted with the finite temporal width of the laser pulses.

## References

[b1] Roncali J., Leriche P. & Blanchard P. Molecular materials for organic photovoltaics: small is beautiful. Adv. Mater. 26, 3821–3838 (2014). 24687246 10.1002/adma.201305999

[b2] Graham K. R. . Importance of the donor: fullerene intermolecular arrangement for high-efficiency organic photovoltaics. J. Amer. Chem. Soc. 136, 9608–9618 (2014). 24932575 10.1021/ja502985g

[b3] Li H. . Beyond fullerenes: design of nonfullerene acceptors for efficient organic photovoltaics. J. Amer. Chem. Soc. 136, 14589–14597 (2014). 25265412 10.1021/ja508472j

[b4] Sai N. . Understanding the interface dipole of copper phthalocyanine (CuPc)/C60: theory and experiment. J. Phys. Chem. Lett. 3, 2173–2177 (2012). 26295767 10.1021/jz300744r

[b5] Ayzner A. L. . Ultrafast electron transfer at organic semiconductor interfaces: importance of molecular orientation. J. Phys. Chem. Lett. 6, 6–12 (2014). 26263084 10.1021/jz502253r

[b6] Rand B. P. . The impact of molecular orientation on the photovoltaic properties of a phthalocyanine/fullerene heterojunction. Adv. Funct. Mater. 22, 2987–2995 (2012).

[b7] Duhm S. . Orientation-dependent ionization energies and interface dipoles in ordered molecular assemblies. Nat. Mater. 7, 326–332 (2008). 18264103 10.1038/nmat2119

[b8] Zhang Y. . Probing carrier transport and structure-property relationship of highly ordered organic semiconductors at the two-dimensional limit. Phys. Rev. Lett. 116, 016602 (2016). 26799035 10.1103/PhysRevLett.116.016602

[b9] Cornil J., Beljonne D., Calbert J. P. & Brédas J. L. Interchain interactions in organic π‐conjugated materials: impact on electronic structure, optical response, and charge transport. Adv. Mater. 13, 1053–1067 (2001).

[b10] Curtis M. D., Cao J. & Kampf J. W. Solid-state packing of conjugated oligomers: From π-stacks to the herringbone structure. J. Amer. Chem. Soc. 126, 4318–4328 (2004). 15053622 10.1021/ja0397916

[b11] Barth J. V. Molecular architectonic on metal surfaces. Annu. Rev. Phys. Chem. 58, 375–407 (2007). 17430091 10.1146/annurev.physchem.56.092503.141259

[b12] Götzen J., Käfer D., Wöll C. & Witte G. Growth and structure of pentacene films on graphite: Weak adhesion as a key for epitaxial film growth. Phys. Rev. B. 81, 085440 (2010).

[b13] Thayer G. E. . Role of surface electronic structure in thin film molecular ordering. Phys. Rev. Lett. 95, 256106 (2005). 16384479 10.1103/PhysRevLett.95.256106

[b14] Verlaak S. . Nucleation of organic semiconductors on inert substrates. Phys. Rev. B. 68, 195409 (2003).

[b15] Li L. . An ultra closely π-stacked organic semiconductor for high performance field-effect transistors. Adv. Mater. 19, 2613–2617 (2007).

[b16] Wagner S. R., Lunt R. R. & Zhang P. Anisotropic crystalline organic step-flow growth on deactivated Si surfaces. Phys. Rev. Lett. 110, 086107 (2013). 23473173 10.1103/PhysRevLett.110.086107

[b17] Wagner S. R. . Growth of metal phthalocyanine on deactivated semiconducting surfaces steered by selective orbital coupling. Phys. Rev. Lett. 115, 096101 (2015). 26371664 10.1103/PhysRevLett.115.096101

[b18] Stadtmüller B., Kröger I., Reinert F. & Kumpf C. Submonolayer growth of CuPc on noble metal surfaces. Phys. Rev. B. 83, 085416 (2011).

[b19] Bobaru S. C., Salomon E., Layet J.-M. & Angot T. Structural properties of iron phtalocyanines on Ag (111): from the submonolayer to monolayer range. J. Phys. Chem. C. 115, 5875–5879 (2011).

[b20] Käfer D., Ruppel L. & Witte G. Growth of pentacene on clean and modified gold surfaces. Phys. Rev. B. 75, 085309 (2007).

[b21] Zheng Y. . Effect of molecule-substrate interaction on thin-film structures and molecular orientation of pentacene on silver and gold. Langmuir 23, 8336–8342 (2007). 17602678 10.1021/la063165f

[b22] Witte G. & Wöll C. Growth of aromatic molecules on solid substrates for applications in organic electronics. J. Mater. Res. 19, 1889–1916 (2004).

[b23] Mativetsky J. M. . Face-on stacking and enhanced out-of-plane hole mobility in graphene-templated copper phthalocyanine. Chem. Commun. 50, 5319–5321 (2014). 10.1039/c3cc47516f 24178059

[b24] Xiao K. . Surface-induced orientation control of CuPc molecules for the epitaxial growth of highly ordered organic crystals on graphene. J. Amer. Chem. Soc. 135, 3680–3687 (2013). 23368998 10.1021/ja3125096

[b25] McAfee T. . Toward single-crystal hybrid-carbon electronics: impact of graphene substrate defect density on copper phthalocyanine film growth. Cryst. Growth Des. 14, 4394–4401 (2014).

[b26] Mao H. Y. . Chemical vapor deposition graphene as structural template to control interfacial molecular orientation of chloroaluminium phthalocyanine. Appl. Phys. Lett. 99, 093301 (2011).

[b27] Chen W. . Molecular orientation-dependent ionization potential of organic thin films. Chem. Mater. 20, 7017–7021 (2008).

[b28] Deng S. & Berry V. Wrinkled, rippled and crumpled graphene: an overview of formation mechanism, electronic properties, and applications. *Mater. Today*, In Press (2015).

[b29] Huang P. Y. . Grains and grain boundaries in single-layer graphene atomic patchwork quilts. Nature 469, 389–392 (2011). 21209615 10.1038/nature09718

[b30] Stadler C. . Tuning intermolecular interaction in long-range-ordered submonolayer organic films. Nat. Phys. 5, 153–158 (2009).

[b31] Mannsfeld S. C. B. & Fritz T. Understanding organic–inorganic heteroepitaxial growth of molecules on crystalline substrates: experiment and theory. Phys. Rev. B. 71, 235405 (2005).

[b32] Stadler C. . Structural investigation of the adsorption of SnPc on Ag (111) using normal-incidence x-ray standing waves. Phys. Rev. B. 74, 035404 (2006).

[b33] Wang T. & Chan W.-L. Dynamical localization limiting the coherent transport range of excitons in organic crystals. J. Phys. Chem. Lett. 5, 1812–1818 (2014). 26273858 10.1021/jz500716k

[b34] Pimpinelli A. & Villain J. Physics of crystal growth. (Cambridge university press, 1998).

[b35] Gopakumar T. G. . Adsorption of palladium phthalocyanine on graphite: STM and LEED study. J. Phys. Chem. B. 108, 7839–7843 (2004).

[b36] Scheffler M. . Structural study of monolayer cobalt phthalocyanine adsorbed on graphite. Surf. Sci. 608, 55–60 (2013).

[b37] Xie W., Xu J., An J. & Xue K. Correlation between molecular packing and surface potential at vanadyl phthalocyanine/HOPG interface. J. Phys. Chem. C. 114, 19044–19047 (2010).

[b38] Wang T. . Effect of interlayer coupling on ultrafast charge transfer from semiconducting molecules to mono-and bilayer graphene. Phys. Rev. Applied 4, 014016 (2015).

[b39] Wang L., Chen W. & Wee A. T. S. Charge transfer across the molecule/metal interface using the core hole clock technique. Surf. Sci. Rep. 63, 465–486 (2008).

[b40] Demetrio Filho A. & Neto P. H. O. Intramolecular reorganization energy in zinc phthalocyanine and its fluorinated derivatives: a joint experimental and theoretical study. Chem. Commun. 49, 6069–6071 (2013). 10.1039/c3cc42003e 23722445

[b41] Bredas J. L., Beljonne D., Coropceanu V. & Cornil J. Charge-Transfer and Energy-Transfer Processes in π-Conjugated Oligomers and Polymers: a Molecular Picture. Chem. Rev. 104, 4971–5004, 10.1021/cr040084k (2004). 15535639

[b42] Lindstrom C. D. & Zhu X.-Y. Photoinduced electron transfer at molecule-metal interfaces. Chem. Rev. 106, 4281–4300 (2006). 17031987 10.1021/cr0501689

[b43] Partoens B. & Peeters F. M. From graphene to graphite: electronic structure around the K point. Phys. Rev. B. 74, 075404 (2006).

[b44] Wei C. . Probing the ultrafast electron transfer at the CuPc/Au (111) interface. Appl. Phys. Lett. 88 (2006).

[b45] Dou W. . Investigation on the orderly growth of thick zinc phthalocyanine films on Ag (100) surface. J. Chem. Phys. 133, 144704 (2010). 20950027 10.1063/1.3489658

[b46] Echenique P. M. . Decay of electronic excitations at metal surfaces. Surf. Sci. Rep. 52, 219–317 (2004).

[b47] Chizhov I., Scoles G. & Kahn A. The influence of steps on the orientation of copper phthalocyanine monolayers on Au (111). Langmuir 16, 4358–4361 (2000).

[b48] Ludwig C. . Epitaxy and scanning tunneling microscopy image contrast of copper–phthalocyanine on graphite and MoS_2_. J. Vac. Sci. Technol. B. 12, 1963–1966 (1994).

[b49] Liu Q. . Metal-catalyst-free and controllable growth of high-quality monolayer and AB-stacked bilayer graphene on silicon dioxide. Carbon 96, 203–211 (2016).

[b50] Li X. . Transfer of large-area graphene films for high-performance transparent conductive electrodes. Nano Lett. 9, 4359–4363 (2009). 19845330 10.1021/nl902623y

