# Supplementary material for: Growing Ultra-flat Organic Films on Graphene with a Face-on Stacking via Moderate Molecule-Substrate Interaction
Source: Sci Rep. 2016 Jun 30;6:28895. doi: 10.1038/srep28895 (PMC4928121; doi:10.1038/srep28895)
Supplement: Supplementary Information [file srep28895-s1.pdf]

Supplementary Information

Growing Ultra-flat Organic Films on Graphene with a Face-on Stacking via Moderate Molecule-Substrate Interaction

Ti Wang[1, *], Tika R. Kafle[1], Bhupal Kattel[1], Qingfeng Liu[1], Judy Wu[1], Wai-Lun Chan[1, *]

[1]Department of Physics and Astronomy, University of Kansas, Lawrence, KS 66045

Corresponding Authors:
*E-mail: wangti@ku.edu (T. W.)
*E-mail: wlchan@ku.edu (W. –L. C.)

1. AFM images with a larger scan size

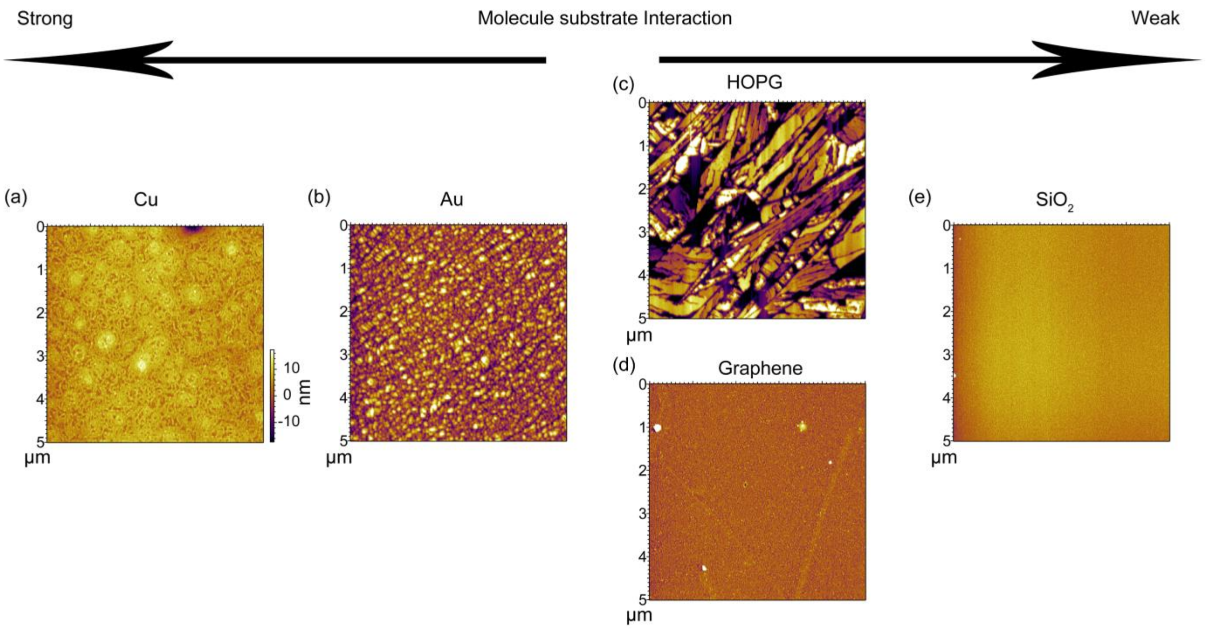

Figure S1: AFM images of 10 nm ZnPc films deposited on (a) Cu, (b) Au, (c) HOPG, (d) graphene and (e) SiO_2. For comparison, the same color scale is used for all the images.

Figure S1 shows the surface morphology measured by AFM for 10 nm ZnPc films deposited on various substrates. The size of the AFM images is 5 μm × 5 μm. The ZnPc thin films have similar morphology at different areas of the sample. Individual grains can be seen more clearly with a smaller scan size (see Fig. 2 in the main text). Island morphology can be found for the

ZnPc films on Cu and Au, while the ZnPc film on Au surface shows a larger grain size. For the ZnPc on HOPG, the surface of each grain is atomically flat, but gaps exist between grains at positions where step-edges on the graphite surface are located. Furthermore, it is apparent from the figure that the surfaces of the ZnPc films on graphene and SiO_2 are very smooth.

2. Island growth for ZnPc on Au

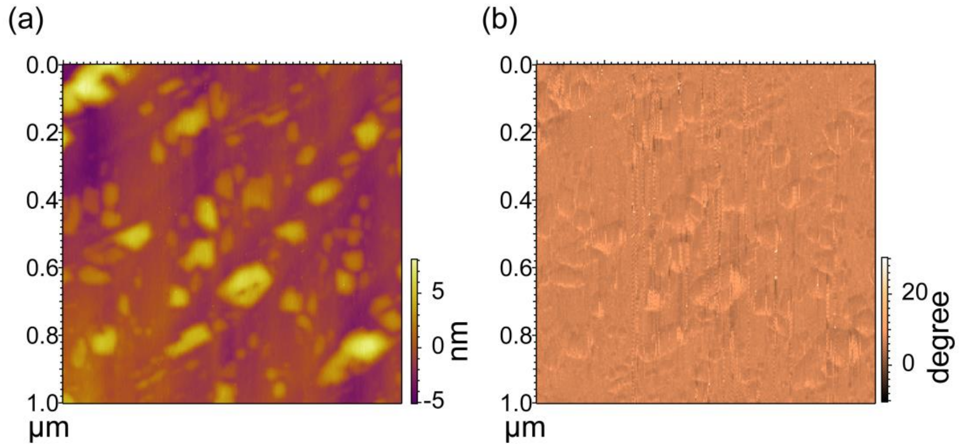

Figure S2: (a) AFM images of 2 nm ZnPc thin films deposited on Au. (b) The AFM phase images of Fig. S2 (a).

Figure S2 (a) and (b) show the AFM image of 2 nm ZnPc on Au and the phase image at the same area, respectively. The phase image indicates that the whole surface has been covered by ZnPc molecules. The AFM image (Fig. S2a) shows that most parts of the film are flat and some small islands form on the surface. It is consistent with the Stranski-Krastanov (SK) growth mode, in which ZnPc molecules grow layer-by-layer initially and then form islands as the film becomes thicker.

3. LEED measurements for 10 nm thick ZnPc films

Figure S3 (a) and (b) show the LEED patterns of 10 nm ZnPc films grown on Au(111) and graphite respectively. The patterns were collected at electron energy equal to 13 eV. The

LEED pattern for ZnPc/Au is similar to those observed for CuPc/Au(111).[1] The pattern corresponds to a face-on stacking.[1] The radius of the innermost ring of diffraction spots can be used to determine the average distance between neighboring molecules within the plane parallel to the surface.[2] The distance calculated from Fig. S3a is 1.40 ± 0.05 nm, which is similar to the value obtained for metal-phthalocyanine molecules with a face-on stacking.[2,3] The ZnPc film on graphite is polycrystalline with a face-on stacking (Fig. S3b). The radius of the first ring in the LEED pattern is used to determine the nearest neighbor distance which is 1.37 ± 0.05 nm.

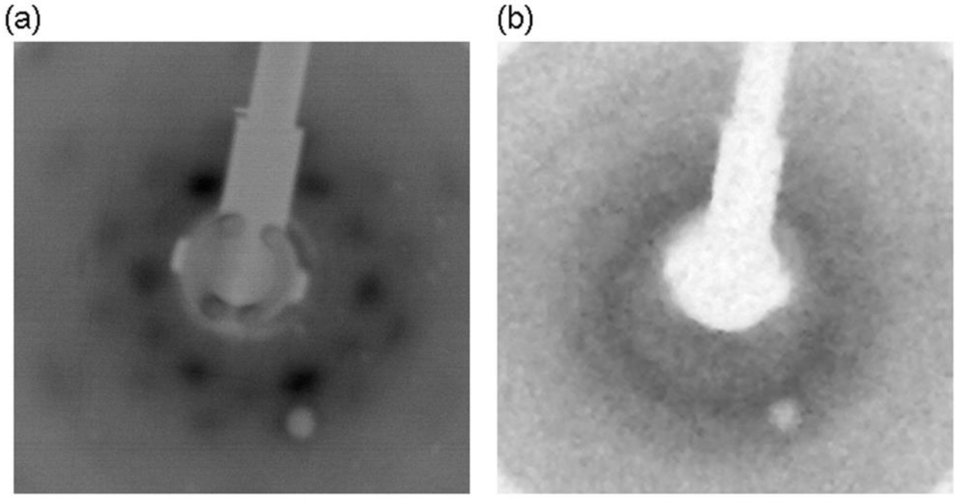

Figure S3: LEED patterns of 10 nm ZnPc (a) on Au and (b) on graphite. Both patterns were collected at electron energy = 13 eV.

References

1 Chizhov, I., Scoles, G. & Kahn, A. The influence of steps on the orientation of copper phthalocyanine monolayers on Au (111). *Langmuir* **16**, 4358-4361 (2000).

2 Stadler, C., Hansen, S., Kröger, I., Kumpf, C. & Umbach, E. Tuning intermolecular interaction in long-range-ordered submonolayer organic films. *Nat. Phys.* **5**, 153-158 (2009).

3 Kröger, I. *et al.* Submonolayer growth of copper-phthalocyanine on Ag (111). *New J. Phys.* **12**, 083038 (2010).
